# Chest CT features of COVID-19 in vaccinated versus unvaccinated patients: use of CT severity score and outcome analysis

**DOI:** 10.1007/s11547-023-01664-z

**Published:** 2023-06-24

**Authors:** Giorgio Maria Masci, Antonella Izzo, Giacomo Bonito, Livia Marchitelli, Elisa Guiducci, Simone Ciaglia, Sonia Lucchese, Laura Corso, Alessandra Valenti, Lucia Malzone, Patrizia Pasculli, Maria Rosa Ciardi, Giuseppe La Torre, Gioacchino Galardo, Francesco Alessandri, Francesco Vullo, Lucia Manganaro, Franco Iafrate, Carlo Catalano, Paolo Ricci

**Affiliations:** 1 Department of Radiological, Oncological and Pathological Sciences, Policlinico Umberto I, Sapienza University of Rome, Viale Regina Elena 324, 00161 Rome, Italy; 2 Unit of Emergency Radiology, Policlinico Umberto I, Sapienza University of Rome, Viale del Policlinico 155, 00161 Rome, Italy; 3 Department of Public Health and Infectious Diseases, Sapienza University of Rome, Piazzale Aldo Moro 5, 00185 Rome, Italy; 4 Medical Emergency Unit, Policlinico Umberto I, Sapienza University of Rome, Viale del Policlinico 155, 00161 Rome, Italy; 5 Department of Anaesthesiology Critical Care Medicine and Pain Therapy, Policlinico Umberto I, Sapienza University of Rome, Viale Regina Elena 324, 00161 Rome, Italy

**Keywords:** COVID-19, SARS-CoV-2, Computed tomography, Pneumonia, Vaccination

## Abstract

**Objectives:**

To evaluate the impact of vaccination on severe acute respiratory syndrome coronavirus 2 (SARS-CoV-2) infection and moreover on coronavirus disease 2019 (COVID-19) pneumonia, by assessing the extent of lung disease using the CT severity score (CTSS).

**Methods:**

Between September 2021 and February 2022, SARS-CoV-2 positive patients who underwent chest CT were retrospectively enrolled. Anamnestic and clinical data, including vaccination status, were obtained. All CT scans were evaluated by two readers using the CTSS, based on a 25-point scale. Univariate and multivariate logistic regression analyses were performed to evaluate the associations between CTSS and clinical or demographic variables. An outcome analysis was used to differentiate clinical outcome between vaccinated and unvaccinated patients.

**Results:**

Of the 1040 patients (537 males, 503 females; median age 58 years), 678 (65.2%) were vaccinated and 362 (34.8%) unvaccinated. Vaccinated patients showed significantly lower CTSS compared to unvaccinated patients (*p* < 0.001), also when patients without lung involvement (CTSS = 0) were excluded (*p* < 0.001). Older age, male gender and lower number of doses administered were associated with higher CTSS, however, in the multivariate analysis, vaccination status resulted to be the variable with the strongest association with CTSS. Clinical outcomes were significantly worse in unvaccinated patients, including higher number of ICU admissions and higher mortality rates.

**Conclusions:**

Lung involvement during COVID-19 was significantly less severe in vaccinated patients compared with unvaccinated patients, who also showed worse clinical outcomes. Vaccination status was the strongest variable associated to the severity of COVID-related, more than age, gender, and number of doses administered.

**Supplementary Information:**

The online version contains supplementary material available at 10.1007/s11547-023-01664-z.

## Introduction

Since the beginning of the coronavirus disease 2019 (COVID-19) pandemic, considerable efforts have been made globally to eradicate the severe acute respiratory syndrome coronavirus 2 (SARS-CoV-2) and to reduce COVID-19-related morbidity and mortality. The main strategy adopted consisted in major investments for the development of vaccines against SARS-CoV-2, and as of September 2022 more than 12 billion doses of vaccine have been administered worldwide [1].

Different types of vaccine have been approved by Food and Drug Administration (FDA) and by European Medicines Agency (EMA), which include mRNA-based vaccines, such as Comirnaty (BNT162b2) produced by Pfizer-BioNTech and Spikevax (mRNA-1273) by Moderna, and viral vector-based vaccines, such as COVID-19 Vaccine Janssen (Ad26.COV2.S) produced by Johnson & Johnson—Janssen and Vaxzevria (ChAdOx1 nCoV-19) by Oxford-AstraZeneca (this last one not approved by the FDA) [2, 3]. Clinical trials have demonstrated the safety and efficacy of these vaccines in protecting against COVID-19 [4–7].

Computed tomography (CT) of the chest is widely considered the best imaging modality for the diagnosis and follow-up of COVID-19-related pneumonia, and several authors have proposed different CT-based scores to assess the severity of lung involvement in SARS-CoV-2 infection [8–12].

In this study, we aimed to investigate the impact of vaccination on the severity of COVID-19 lung disease by the assessment and comparison of pulmonary involvement in vaccinated and unvaccinated SARS-CoV-2 patients using a previously described CT-based scoring system.

## Materials and methods

### Study design

This single-center retrospective observational study was conducted on consecutive patients with clinical suspicion for SARS-CoV-2 infection, who underwent chest CT scan in the Unit of Emergency Radiology of our Institution, between September 2021 and February 2022, and with subsequent confirmed positive reverse transcription–polymerase chain reaction (RT-PCR) for SARS-CoV-2 performed the same day of CT.

Clinical suspicion was established according to the Global surveillance for COVID-19 by the World Health Organization [13].

The appropriateness of performing CT examination was established on the basis of a series of clinical parameters and by the clinical team’s judgement, as indicated by the Fleischner Society [14]. The clinical workflow of our hospital for the decision-making process is represented in the supplemental figure.

Anamnestic and clinical information regarding demographics, comorbidities, laboratory findings and vaccination data, including the type of vaccine and the number of doses received, were collected from the electronic medical records; patients with no available vaccination data or without laboratory findings were excluded from the study.

The study was approved by the local ethics committee (protocol number 298/2020), which waived the need of written informed consent due to the retrospective nature of the study.

### CT protocol and image analysis

A standard high-resolution CT of the thorax was performed in all cases with a multidetector CT scanner (Somatom Sensation 64; Siemens Healthineers) without intravenous contrast injection, except in case of suspicion of pulmonary embolism. Images were then reconstructed with a 1-mm slice thickness both with a soft tissue kernel (B20) and a lung kernel (B60) on axial, coronal and sagittal planes.

All CT examinations were evaluated by two readers, one radiology resident at the last year of training (junior reader) and one senior radiologist with over 20 years of experience in thoracic imaging (senior reader), blinded to the clinical data of patient, including the vaccination status. A semi-quantitative CT Severity Score (CTSS) was calculated for all patients considering the extent of parenchymal involvement per each of the 5 lobes: 0, no involvement; 1, < 5% involvement; 2, 5–25% involvement; 3, 26–50% involvement; 4, 51–75% involvement; 5, > 75% involvement. The sum of each lobar score resulted in the CTSS, ranging from 0 to 25 [10].

The main pattern of lung opacities was also described for each patient, according to the standard glossary for thoracic imaging reported by the Fleischner Society: ground glass opacity (GGO), crazy-paving pattern or pulmonary consolidation [15].

Pleural effusion and lymphadenopathies (short axis > 10 mm) were described when present.

### Statistical analysis

Data were analyzed using statistical software (SPSS version 25.0, IBM Corp). Continuous variables were expressed as median value and interquartile range (IQR). The frequencies of demographic and clinical characteristics of populations were expressed as the number (percentage) of occurrences and were compared using the 2-tailed χ2 test or Fisher’s exact test. The Mann–Whitney test was used for single comparisons. Univariate, bivariate and multivariate logistic regression were performed to identify relationships between the CTSS and independent variables (age, gender, vaccination status and number of doses received) and for the outcome analysis. Differences for which *p* < 0.05 were considered statistically significant.

The interobserver agreement between the two readers for CTSS assessment was calculated using the intraclass correlation coefficient (ICC).

## Results

### Study population

Between September 1st 2021 and February 28th 2022, a total of 1630 patients with a positive RT-PCR result for SARS-CoV-2 underwent a chest CT scan and were enrolled for this study. The median turnaround time (TAT) for RT-PCR results was 6.2 h [4.9, 7.7]. Laboratory findings or vaccination data were not available in 522 patients, while imaging artifacts not allowing a precise assessment of lung parenchyma were observed in 68 cases: these 590 patients were therefore excluded from the study.

**Fig. 1:**
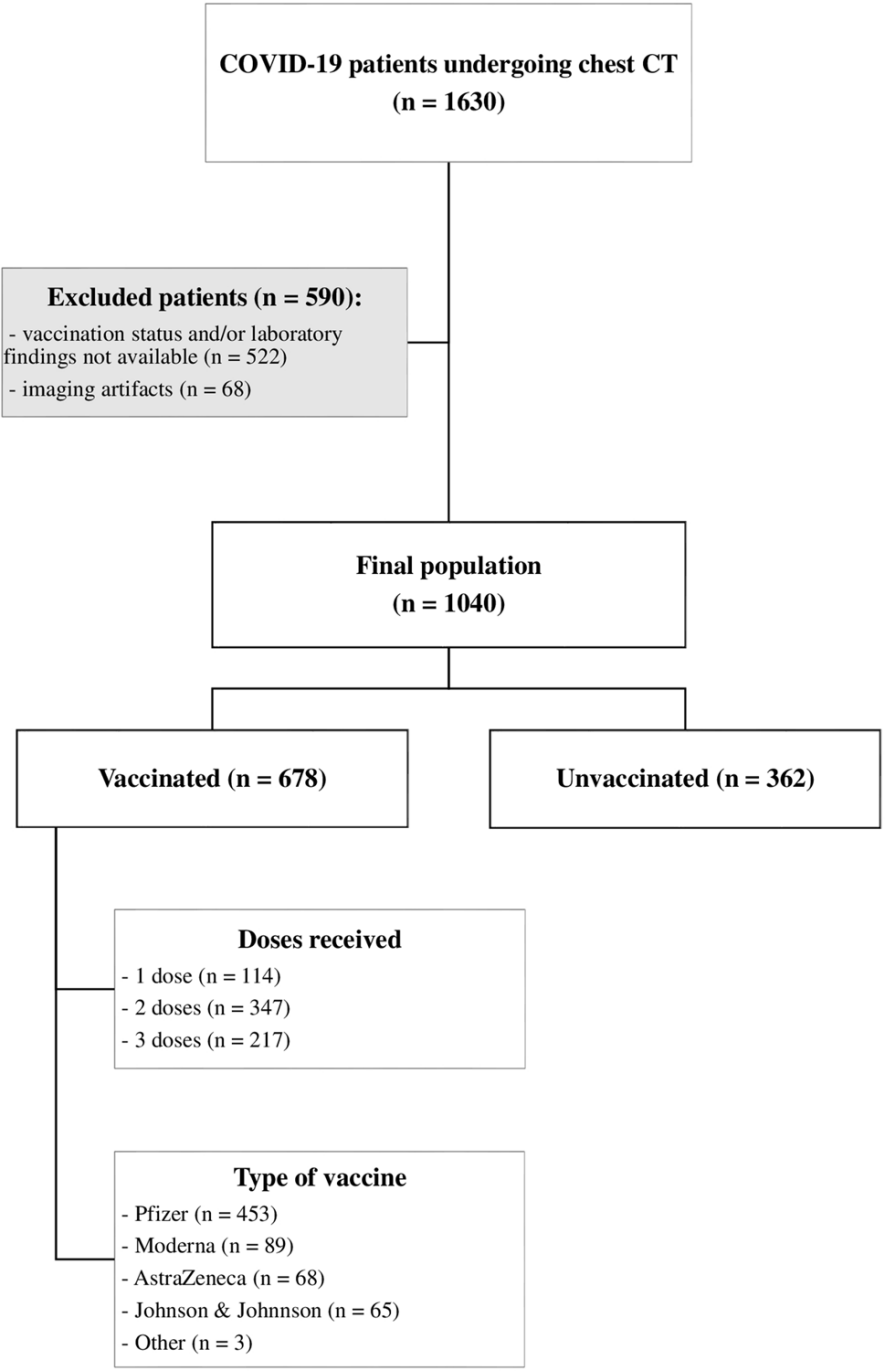
Flowchart of the study population

Final population consisted of 1040 patients (537 males, 503 females; median age 58 [43, 73]), 678 of which were vaccinated and 362 were not vaccinated against COVID-19. Among vaccinated patients, 114 (16.8%) received 1 dose of vaccine, 347 (51.2%) received 2 doses, and 217 (32%) were vaccinated with 3 doses. Regarding the type of vaccine received, 453 (66.8%) patients were vaccinated with Comirnaty (Pfizer-BioNTech), 89 (13.1%) with Spikevax (Moderna), 68 (10%) with Vaxzevria (Oxford-AstraZeneca), 65 (9.6%) with Janssen (Johnson & Johnson–Janssen), and 3 (0.5%) patients received other vaccines than those approved by EMA. A flow-chart of our study population is represented in Fig. 1.

### Demographic and clinical characteristics by vaccination status

Of the 678 vaccinated patients, 353 were male and 325 female, and median age was 60 years [42, 74], while among the 362 unvaccinated patients, 184 were male and 178 female, and median age was 56 years [44, 71]. In both groups, most patients presented with fever, cough, and dyspnea. The median time interval between symptom’s onset and CT scan was 6 days [5, 7] days for vaccinated patients and 6 days [4, 7] days for unvaccinated patients (*p* = 0.216). Leukopenia (WBC < 4000/mm^3^) was found in 91/678 (13.4%) vaccinated patients and in 42/362 (11.6%) unvaccinated patients (*p* = 0.436); increased CRP levels (> 0.5 mg/dL) were found in 583/678 (86%) vaccinated patients and in 329/362 (90.9%) unvaccinated patients (*p* = 0.023); decreased PaO2/FiO2 ratio (< 300) was observed in 197/678 (29.1%) vaccinated patients and in 159/362 (43.9%) unvaccinated patients (*p* < 0.001); increased D-dimer levels (> 500 ng/mL) were found in 461/678 (68%) vaccinated patients and in 316/362 (87.3%) unvaccinated patients (*p* < 0.001); increased LDH levels (> 300 U/l) were found in 472/678 (69.6%) vaccinated patients and in 268/362 (74%) unvaccinated patients (*p* = 0.151).

Main comorbidities were hypertension, obesity, diabetes, and chronic obstructive pulmonary disease (COPD), but no significant differences in frequency were observed between vaccinated and unvaccinated patients.

Demographic and clinical characteristics of the two groups are summarized in Table 1.

**Table 1:** Characteristics of study population

Variable	All patients (n = 1040)	Vaccinated (n = 678)	Unvaccinated (n = 362)
Gender			
Male	537 (51.6%)	353 (52.1%)	184 (50.8%)
Female	503 (48.4%)	325 (47.9%)	178 (49.2%)
Age *	58 [43, 73]	60 [42, 74]	56 [44, 71]
Symptoms			
Fever	795 (76.4%)	499 (73.6%)	296 (81.8%)
Cough	708 (68.1%)	452 (67.7%)	256 (70.7%)
Dyspnea	386 (37.1%)	191 (28.2%)	195 (53.9%)
Clinical and laboratory findings			
Leukopenia	133 (12.8%)	91 (13.4%)	42 (11.6%)
Increased CRP level	912 (87.7%)	583 (86%)	329 (90.9%)
Decreased PaO_2_/FiO_2_ ratio	356 (34.2%)	197 (29.1%)	159 (43.9%)
Increased D-dimer level	777 (74.7%)	461 (68%)	316 (87.3%)
Increased LDH level	740 (71.2%)	472 (69.6%)	268 (74%)
Comorbidities			
Hypertension	352 (33.8%)	237 (35%)	115 (31.8%)
Diabetes	229 (22%)	138 (20.4%)	91 (25.1%)
Chronic obstructive pulmonary disease	117 (11.3%)	72 (10.6%)	45 (12.4%)
Obesity	288 (27.7%)	193 (28.5%)	95 (26.2%)
No. of doses			
0	362 (34.8%)	–	362 (100%)
1	114 (11%)	114 (16.8%)	–
2	347 (33.4%)	347 (51.2%)	–
3	217 (20.8%)	217 (32%)	–
Type of vaccine			
Pfizer-BioNTech	453 (43.5%)	453 (66.8%)	–
Moderna	89 (8.6%)	89 (13.1%)	–
AstraZeneca	68 (6.5%)	68 (10%)	–
Johnson & Johnson	65 (6.3%)	65 (9.6%)	–
Other	3 (0.3%)	3 (0.5%)	–
None	362 (34.8%)	–	362 (100%)

Except where indicated, data are expressed as number of patients (percentage)

*Data are expressed as median value [interquartile range (IQR)]

### Assessment of CT severity score (CTSS) and CT features

The interobserver variability analysis for the assessment of CTSS showed good agreement between junior and senior readers, with an ICC of 0.884. The specified findings, according with both junior and senior readers, are displayed in Tables 2 and 3.

**Table 2:** Univariate analysis

Variable	CTSS (junior reader)	CTSS (senior reader)
Vaccination status (all patients)		
Unvaccinated	10 [2.3, 14]	9 [1, 15]
Vaccinated	1 [0, 9]	0 [0, 8]
*p*	< 0.001	< 0.001
Vaccination status (only patients with CTSS > 0)		
Unvaccinated	11 [8, 15]	11 [7, 16]
Vaccinated	8 [4, 13]	8 [4, 13]
*p*	< 0.001	< 0.001
Gender		
Male	7 [0, 12]	5 [0, 12]
Female	2 [0, 10]	2 [0, 9]
*P*	< 0.001	< 0.001

Data are expressed as median value [interquartile range (IQR)]. *CTSS* CT severity score

**Table 3:** Bivariate analysis

	CTSS (junior reader)	CTSS (senior reader)	Age	No. of doses
CTSS (junior reader)	–	r = 0.802 *p* < 0.001	r = 0.409 *p* < 0.001	r = − 0.332 *p* < 0.001
CTSS (senior reader)	r = 0.802 *p* < 0.001	–	r = 0.303 *p* < 0.001	r = − 0.354 *p* < 0.001
Age	r = 0.409 *p* < 0.001	r = 0.303 *p* < 0.001	–	r = 0.066 *p* = 0.044
No. of doses	r = − 0.332 *p* < 0.001	r = − 0.354 *p* < 0.001	r = 0.066 *p* = 0.044	–

*CTSS* CT severity score

**Fig. 2:**
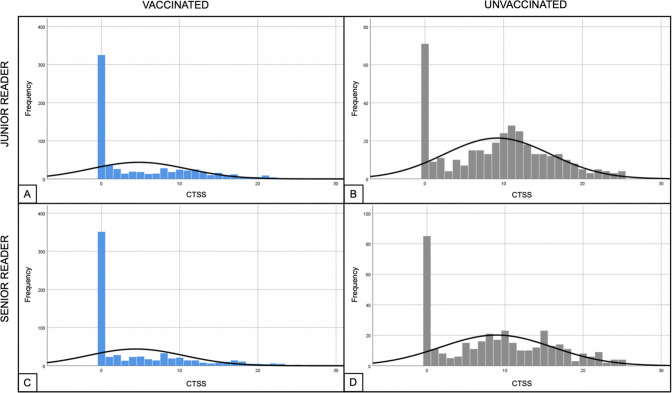
Comparison of frequencies of CTSS between vaccinated and unvaccinated patients, considering all CTSS

According to senior reader, the CTSS resulted significantly lower in vaccinated patients compared to unvaccinated patients (*p* < 0.001), with a median value of 0 [0, 8] in vaccinated patients and 9 [1, 15] in unvaccinated patients (Table 2 and Fig. 2).

**Fig. 3:**
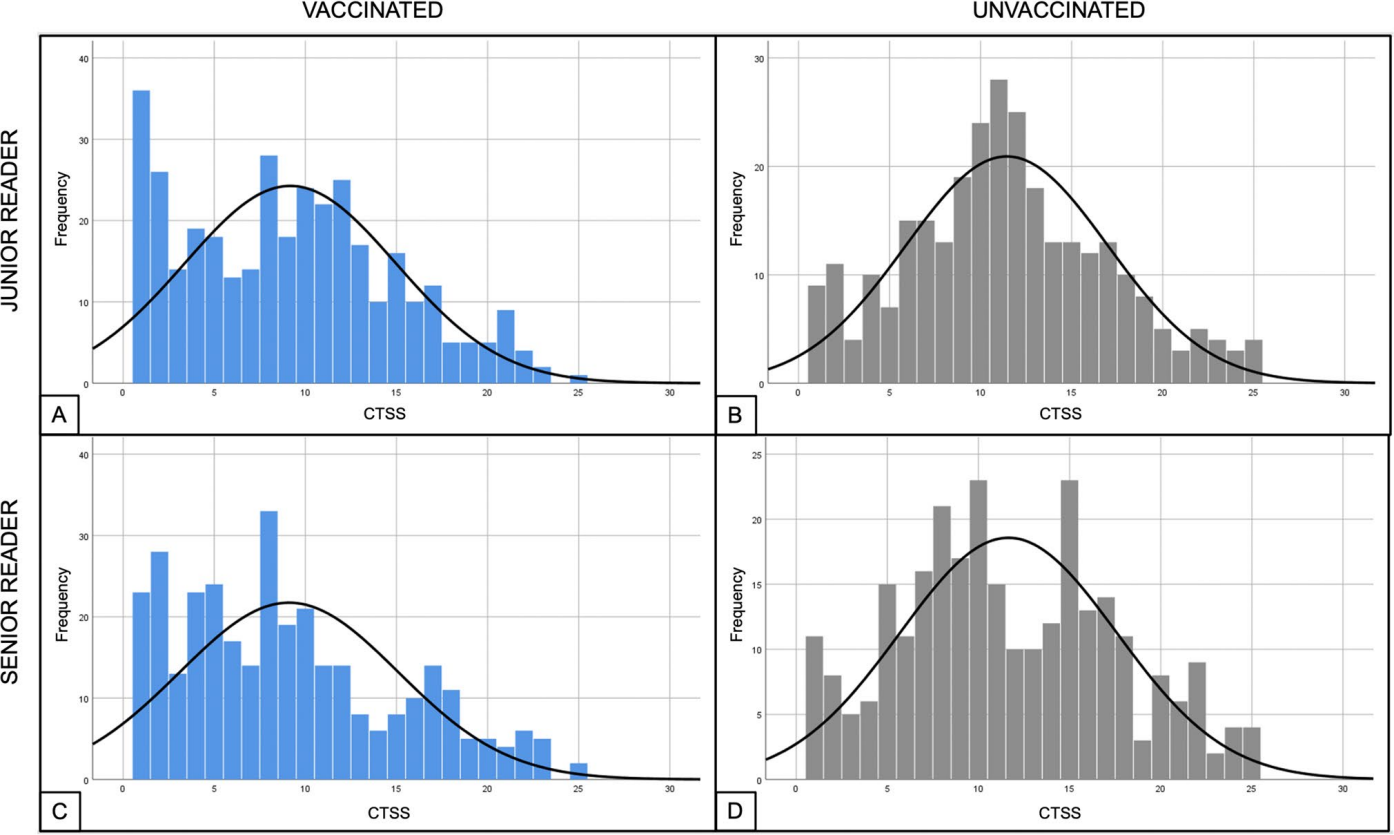
Comparison of frequencies of CTSS between vaccinated and unvaccinated patients, considering only CTSS > 0

When excluding patients without lung involvement (CTSS = 0) the statistical difference between the two groups was confirmed (*p* < 0.001), with a median CTSS of 8 [4, 13] in vaccinated patients and 11 [7, 16] in unvaccinated patients (Table 2 and Fig. 3).

When parenchymal involvement was compared between genders, male patients showed higher CTSS compared to females with a median value of 5 [0, 12] and 2 [0, 9] (*p* < 0.001) (Table 2).

A bivariate analysis was performed to identify the association of CTSS with independent variables such as age and number of doses received (Table 3). CTSS showed a positive linear correlation with age (r = 0.303, *p* < 0.001) and a negative linear correlation with number of doses received (r = -0.354, *p* < 0.001).

The most frequent pattern was GGO both in vaccinated and unvaccinated patients, followed by crazy-paving and consolidation; GGO and crazy-paving patterns proved to be more prevalent among vaccinated patients (*p* = 0.001).

No differences were found between vaccinated and unvaccinated patients regarding the presence of pleural effusion and enlarged lymph nodes.

### Multivariate regression analysis considering vaccination status, age, gender, and number of doses received

**Fig. 4:**
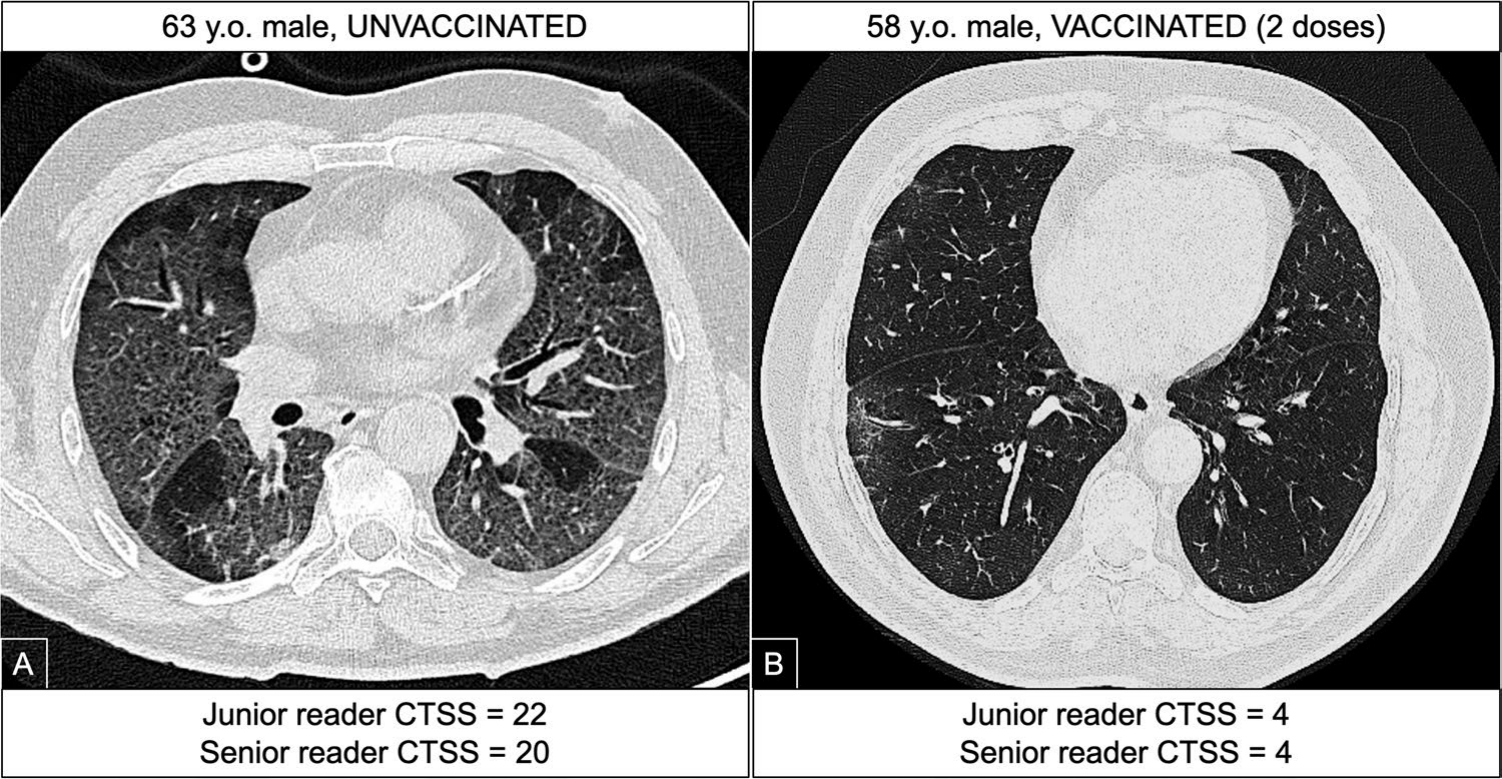
Case of a 63-year-old unvaccinated patient with bilateral interstitial pneumonia (**A**) and a 58-year-old vaccinated patient with small and focal ground glass opacities (**B**)

A multivariate regression analysis was performed using CTSS as dependent variable with two different models: the first one considering vaccination status, age, and gender; the second model included age, gender, and number of doses received. The analysis was conducted considering CTSS both according to junior and senior reading. Vaccination status was found to be the variable with the highest B and β coefficients for both readers, also when only patients with parenchymal involvement (CTSS > 0) were considered (Fig. 4). The results with corresponding regression coefficients of each variable are represented in Table 4.

**Table 4:** Multivariate regression analysis

Variable	Junior reader					Senior reader				
	R^2^	B	St. Err	β	*p*	R^2^	B	St. Err	β	*p*
Model 1 (any CTSS)	0.273					0.198				
Vaccination		− 4.540	0.375	− 0.321	< 0.001		− 4.620	0.402	− 0.320	< 0.001
Age		0.136	0.009	0.395	< 0.001		0.101	0.010	0.286	< 0.001
Gender (F)		− 2.103	0.357	− 0.156	< 0.001		− 2.031	0.383	− 0.148	< 0.001
Model 2 (any CTSS)	0.282					0.219				
Age		0.144	0.010	0.411	< .001		0.109	0.010	0.303	< 0.001
Gender (F)		− 1.932	0.379	− 0.143	< .001		− 1.984	0.404	− 0.143	< 0.001
No. of doses		− 1.913	0.160	− 0.335	< .001		− 2.034	0.170	− 0.349	< 0.001
Model 1 (CTSS > 0)	0.102					0.098				
Vaccination		− 2.915	0.447	− 0.251	< .001		− 3.791	0.513	− 0.285	< 0.001
Age		0.080	0.013	0.234	< .001		0.050	0.015	0.127	0.001
Gender (F)		− 1.666	0.441	− 0.143	< .001		− 2.153	0.507	− 0.161	< 0.001
Model 2 (CTSS > 0)	0.112					0.128				
Age		0.092	0.014	0.268	< .001		0.065	0.016	0.167	< .001
Gender (F)		− 1.825	0.460	− 0.157	< .001		− 2.426	0.523	− 0.181	< 0.001
No. of doses		− 1.258	0.205	− 0.249	< .001		− 1.906	0.233	− 0.329	< 0.001

CT Severity Score (CTSS) was used as dependent variable. *F* female

### Outcome analysis: patients’ discharge, respiratory support, ICU admission and deaths

In total, 433 patients did not receive any medical assistance and were discharged on the same day of CT examination, 339/678 (50%) among vaccinated patients and 94/362 (26%) out of unvaccinated patients.

**Fig. 5:**
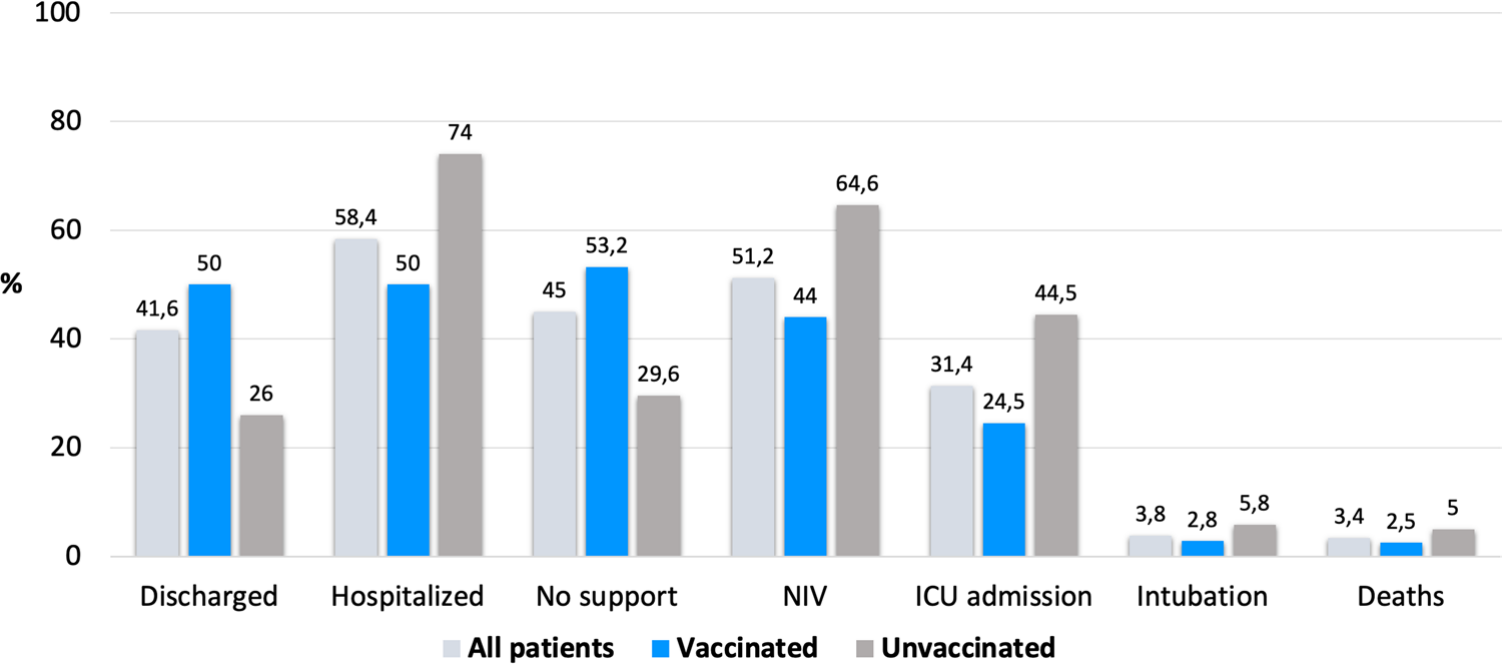
Chart of the outcome analysis of our population

Of the remaining hospitalized patients, most vaccinated ones did not need any respiratory support (298/678, 44%) whilst most unvaccinated patients (234/362, 64.6%) required at least non-invasive ventilation (NIV) (Fig. 5 and supplemental table).

During hospitalization, 327 patients were admitted to the intensive care unit (ICU), 166/678 (24.5%) of which were vaccinated and 161/362 (44.5%) were not vaccinated. Among these patients, orotracheal intubation was needed in 19 vaccinated patients (2.8%), 17 (2.5%) of which died, and in 21 (5.8%) unvaccinated patients, 18 (5%) of which died.

In the multivariate regression analysis higher CTSS were significantly associated with death both in junior reading (B = 0.081, *p* = 0.006) and senior reading (B = 0.051, *p* = 0.036), while the admission to ICU was significantly associated to age (B = 0.011, *p* = 0.002).

Data of the outcome analysis are represented in Fig. 5 and are reported in the supplemental table.

## Discussion

The main purpose of our study was to investigate whether the severity of COVID-19 pneumonia differed between vaccinated and unvaccinated patients using a previously validated semi-quantitative CT-based scoring system, the CT severity score (CTSS), to assess the extent of lung disease in SARS-CoV-2 + patients [10, 16].

CTSS was found to be significantly higher in unvaccinated patients compared to vaccinated patients (*p* < 0.001), showing a considerably greater severity of lung disease in unvaccinated patients.

Moreover, since a large part of vaccinated patients did not show any COVID-related lung abnormalities and were scored as zero, the same comparison of CTSS between the two groups was conducted including only patients with a CTSS > 0; this analysis also confirmed the previous results, showing that also considering only patients with parenchymal involvement, unvaccinated patients had higher CTSS compared to vaccinated patients (*p* < 0.001).

These findings are consistent with the two research studies – the only ones, to the best of our knowledge – that compared the imaging findings of COVID-19 pneumonia between vaccinated and unvaccinated patients [17, 18]. However, Lee et al. [17] conducted a multicenter cohort study on 761 patients with a 2-point scale score based on chest radiographs, 412 of which underwent also CT examination; we believe that our scoring system, due to its larger scale, may help stratifying patients more accurately based on the severity of pneumonia. Vicini et al. [18] performed a single-center analysis on 467 patient who underwent CT scan, comparing fully vaccinated and unvaccinated patients with the same scoring system we used, ranging from 0 to 25 points; in this case, our study, which was performed on a larger sample population, adds important information on the minor strength of other independent variables compared to vaccination status and provides a detailed outcome analysis of our population differentiating vaccinated from unvaccinated patients.

Higher values of CTSS were found to be associated with some demographic characteristics, such as male gender and older age, confirming what previously observed in other studies [19–21]. Moreover, an increase of received vaccine doses was associated with decreasing CTSS, meaning that patients who received more vaccine doses tend to present with a mild disease, in accordance to the evidence reported by recent clinical analyses [22–24].

All significant variables (vaccination status, gender, age, and number of doses) were included in a multivariate regression analysis for identifying the one with the strongest association with CTSS, using two different models (Table 4). Vaccination status was found to be the variable with the strongest association with CTSS, meaning that vaccination was significantly associated with lower CTSS and therefore with less severe forms of pneumonia. The same multivariate regression analysis was also conducted including only patients with CT evidence of pneumonia (CTSS > 0) and also in this case vaccination status was the variable with the strongest association with CTSS.

This data further supports the efficacy of vaccination against COVID-19, as also demonstrated by recent clinical and trial studies [25–27].

The most common pattern was GGO, both in vaccinated and unvaccinated patients, reflecting the acute or subacute phase in which CT was performed.

An outcome analysis of our population was performed considering patients’ discharge, requirement for respiratory support, admission to ICU and deaths (Fig. 5 and supplemental table): most of the patients who were discharged on the same day of CT scan were vaccinated, whereas the majority of hospitalized patients were not vaccinated; moreover, unvaccinated patients showed a greater need for respiratory support (which included both NIV and orotracheal intubation) compared to vaccinated patients. Most of the patients admitted to ICU were not vaccinated, and they also showed a higher mortality rate compared to vaccinated patients.

In the regression analysis no significant correlation was found between vaccination status and the outcome of patients (death or admission to ICU); however, mortality was associated with higher CTSS, while admission to ICU was associated with age. These results highlight how vaccination status alone may not directly be associated with patient’s outcome, but it has to be considered together with the severity of pneumonia (indicated by CTSS) and the age of patients.

The results regarding the clinical outcome of our patients are in accordance with other clinical studies that provided evidence of the efficacy of vaccination against COVID-19 [22, 28, 29].

This study has some limitations. First, we did not consider the time frame between the last vaccination and the date of CT examination, as well as eventual previous SARS-CoV-2 infection, which could both affect the severity of COVID-19-related pneumonia due to their effect on the immune system and on the level of antibodies.

Moreover, patients were not categorized on the basis of timing of symptoms’ onset, which could have been useful to differentiate the severity of lung pneumonia; however, the time interval between symptoms’ onset and CT scan did not differ between vaccinated and unvaccinated patients, therefore we believe that this does not impact the main study’s result.

Finally, the prevalence of SARS-CoV-2 variants among our population was not analyzed, although we believe that the predominant variants during the observational period of our study were Delta (B.1.617.2) and Omicron (B.1.1.529).

In conclusion, this study demonstrates that vaccination against SARS-CoV-2 was associated with less severe COVID-19 pneumonia with milder lung disease and better outcome of vaccinated patients compared to unvaccinated patients, which showed greater parenchymal involvement and higher mortality rates. Moreover, younger age, female gender and higher number of vaccine doses received were also associated with less severe lung disease, but the presence of vaccination remains the most protective factor against COVID-19 pneumonia.

## Supplementary Information

Below is the link to the electronic supplementary material.

Supplementary file1 (DOCX 14704 KB)

## Abbreviations

COVID-19: Coronavirus disease 2019
SARS-CoV-2: Severe acute respiratory syndrome coronavirus 2
CTSS: CT severity score
RT-PCR: Reverse transcriptase polymerase chain reaction
CRP: C-reactive protein
LDH: Lactate dehydrogenase
ICU: Intensive care unit

## References

[1] WHO Coronavirus (COVID-19) Dashboard. https://covid19.who.int. Accessed 6 Nov 2022

[2] Commissioner O of the (2022) COVID-19 Vaccines. FDA

[3] EMA (2021) COVID-19 vaccines: authorised. In: European Medicines Agency. https://www.ema.europa.eu/en/human-regulatory/overview/public-health-threats/coronavirus-disease-covid-19/treatments-vaccines/vaccines-covid-19/covid-19-vaccines-authorised. Accessed 27 Mar 2022

[4] Moreira ED, Kitchin N, Xu X (2022). Safety and efficacy of a third dose of BNT162b2 Covid-19 vaccine. N Engl J Med.

[5] Baden LR, El Sahly HM, Essink B (2021). Efficacy and safety of the mRNA-1273 SARS-CoV-2 vaccine. N Engl J Med.

[6] Sadoff J, Gray G, Vandebosch A (2022). Final analysis of efficacy and safety of single-dose Ad26.COV2.S. N Engl J Med.

[7] Falsey AR, Sobieszczyk ME, Hirsch I (2021). Phase 3 safety and efficacy of AZD1222 (ChAdOx1 nCoV-19) Covid-19 vaccine. N Engl J Med.

[8] Study of Thoracic CT in COVID-19: The STOIC Project—PubMed. https://pubmed.ncbi.nlm.nih.gov/34184935/. Accessed 27 Mar 2022

[9] Han X, Fan Y, Alwalid O (2021). Six-month follow-up chest CT findings after severe COVID-19 pneumonia. Radiology.

[10] Francone M, Iafrate F, Masci GM (2020). Chest CT score in COVID-19 patients: correlation with disease severity and short-term prognosis. Eur Radiol.

[11] Chest CT in COVID-19 at the ED: validation of the COVID-19 reporting and data system (CO-RADS) and CT severity score—CHEST. https://journal.chestnet.org/article/S0012-3692(20)35311-3/fulltext. Accessed 27 Mar 2022

[12] Pan F, Ye T, Sun P (2020). Time course of lung changes on chest CT during recovery from 2019 novel Coronavirus (COVID-19) pneumonia. Radiology.

[13] Organization WH (2020). Global surveillance for COVID-19 caused by human infection with COVID-19 virus: interim guidance, 20 March 2020.

[14] Rubin GD, Ryerson CJ, Haramati LB (2020). The role of chest imaging in patient management during the COVID-19 pandemic: a multinational consensus statement from the Fleischner Society. Chest.

[15] Hansell DM, Bankier AA, MacMahon H (2008). Fleischner society: glossary of terms for thoracic imaging. Radiology.

[16] Pasculli P, Zingaropoli MA, Masci GM (2021). Chest computed tomography score, cycle threshold values and secondary infection in predicting COVID-19 mortality. New Microbiol.

[17] Lee JE, Hwang M, Kim Y-H (2022). Imaging and clinical features of COVID-19 breakthrough infections: a multicenter study. Radiology.

[18] Vicini S, Bellini D, Iannarelli A (2022). Pneumonia frequency and severity in patients with symptomatic COVID-19: impact of mRNA and adenovirus vector vaccines. AJR Am J Roentgenol.

[19] Gao Y, Ding M, Dong X (2021). Risk factors for severe and critically ill COVID-19 patients: a review. Allergy.

[20] Zhang J, Wang X, Jia X (2020). Risk factors for disease severity, unimprovement, and mortality in COVID-19 patients in Wuhan, China. Clin Microbiol Infect.

[21] Zhou F, Yu T, Du R (2020). Clinical course and risk factors for mortality of adult inpatients with COVID-19 in Wuhan, China: a retrospective cohort study. Lancet.

[22] Barda N, Dagan N, Cohen C (2021). Effectiveness of a third dose of the BNT162b2 mRNA COVID-19 vaccine for preventing severe outcomes in Israel: an observational study. Lancet.

[23] Bar-On YM, Goldberg Y, Mandel M (2021). Protection of BNT162b2 vaccine booster against Covid-19 in Israel. N Engl J Med.

[24] Patalon T, Gazit S, Pitzer VE (2022). Odds of testing positive for SARS-CoV-2 following receipt of 3 vs 2 doses of the BNT162b2 mRNA vaccine. JAMA Intern Med.

[25] Polack FP, Thomas SJ, Kitchin N (2020). Safety and efficacy of the BNT162b2 mRNA Covid-19 vaccine. N Engl J Med.

[26] Voysey M, Clemens SAC, Madhi SA (2021). Safety and efficacy of the ChAdOx1 nCoV-19 vaccine (AZD1222) against SARS-CoV-2: an interim analysis of four randomised controlled trials in Brazil, South Africa, and the UK. Lancet.

[27] Voysey M, Costa Clemens SA, Madhi SA (2021). Single-dose administration and the influence of the timing of the booster dose on immunogenicity and efficacy of ChAdOx1 nCoV-19 (AZD1222) vaccine: a pooled analysis of four randomised trials. Lancet.

[28] Lopez Bernal J, Andrews N, Gower C (2021). Effectiveness of the Pfizer-BioNTech and Oxford-AstraZeneca vaccines on covid-19 related symptoms, hospital admissions, and mortality in older adults in England: test negative case-control study. BMJ.

[29] Tartof SY, Slezak JM, Fischer H (2021). Effectiveness of mRNA BNT162b2 COVID-19 vaccine up to 6 months in a large integrated health system in the USA: a retrospective cohort study. Lancet.

