# Supplementary material for: Chest CT features of COVID-19 in vaccinated versus unvaccinated patients: use of CT severity score and outcome analysis
Source: Radiol Med. 2023 Jun 24;128(8):934–43. doi: 10.1007/s11547-023-01664-z (PMC10338619; doi:10.1007/s11547-023-01664-z)
Supplement: Supplementary file 1 — Supplementary file1 (DOCX 14704 KB) [file 11547_2023_1664_MOESM1_ESM.docx]

Supplemental Table. Outcome analysis.

| Variable | All patients (n=1040) | Vaccinated (n=678) | Unvaccinated (n=362) | p value |
| --- | --- | --- | --- | --- |
| Discharge on the same day of CT | 433 (41.6%) | 339 (50%) | 94 (26%) | < .001 |
| Hospitalized patients | 607 (58.4%) | 339 (50%) | 268 (74%) | < .001 |
| Respiratory support |  |  |  |  |
| None | 468 (45%) | 361 (53.2%) | 107 (29.6%) | < .001 |
| NIV | 532 (51.2%) | 298 (44%) | 234 (64.6%) | < .001 |
| Intubation | 40 (3.8%) | 19 (2.8%) | 21 (5.8%) | .026 |
| ICU admission | 327 (31.4%) | 166 (24.5%) | 161 (44.5%) | < .001 |
| Deaths | 35 (3.4%) | 17 (2.5%) | 18 (5%) | .046 |

*NIV: non-invasive ventilation. ICU: intensive care unit.*

Supplemental Figure. Workflow of patients’ management at our Institution


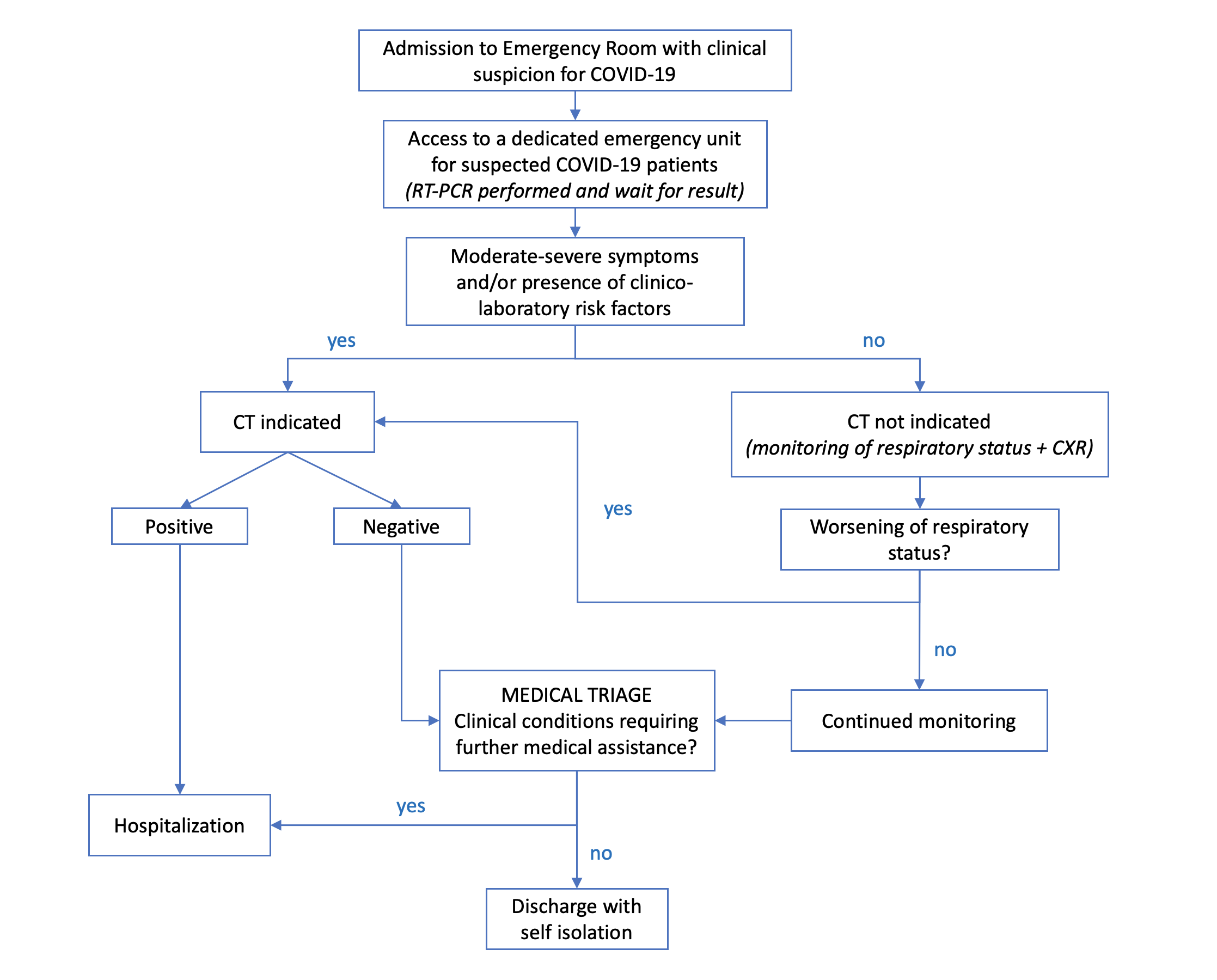


*RT-PCR: reverse transcriptase polymerase chain reaction. CXR: chest x-ray.*
